# A novel start-loss mutation of the *SLC29A3* gene in a consanguineous family with H syndrome: clinical characteristics, in silico analysis and literature review

**DOI:** 10.1186/s12920-024-01949-w

**Published:** 2024-07-04

**Authors:** Nahid Rezaie, Nader Mansour Samaei, Ayda Ghorbani, Naghmeh Gholipour, Shohreh Vosough, Mahboobeh Rafigh, Abolfazl Amini

**Affiliations:** 1 Department of Medical Genetics, School of Advanced Technologies in Medicine, Golestan University of Medical Sciences, Gorgan, Iran; 2 Gorgan Congenital Malformations Research Center, Golestan University of Medical Sciences, Gorgan, Iran; 3 Department of Cytogenetics, Genome Genetics Laboratory, Gorgan, Golestan Iran; 4 Department of Molecular Genetics, Faculty of Biological Science, Tarbiat Modares University, Tehran, Iran; 5 Department of Obstetrics and Gynecology, School of Medicine, Sayyad Shirazi Hospital, Golestan University of Medical Sciences, Gorgan, Iran; 6 Medical Genetics Research Center, Faculty of Medicine, Mashhad University of Medical Sciences, Mashhad, Iran; 7 Laboratory Sciences Research Center, Golestan University of Medical Sciences, Gorgan, Iran; 8 Department of Medical Biotechnology, Faculty of Advanced Technologies in Medicine, Golestan University of Medical Sciences, Gorgan, Iran

**Keywords:** H syndrome, *SLC29A3* gene, Novel mutation, Hyperpigmentation, Whole-exome sequencing, Iran

## Abstract

**Background:**

The *SLC29A3* gene, which encodes a nucleoside transporter protein, is primarily located in intracellular membranes. The mutations in this gene can give rise to various clinical manifestations, including H syndrome, dysosteosclerosis, Faisalabad histiocytosis, and pigmented hypertrichosis with insulin-dependent diabetes. The aim of this study is to present two Iranian patients with H syndrome and to describe a novel start-loss mutation in *SLC29A3* gene.

**Methods:**

In this study, we employed whole-exome sequencing (WES) as a method to identify genetic variations that contribute to the development of H syndrome in a 16-year-old girl and her 8-year-old brother. These siblings were part of an Iranian family with consanguineous parents. To confirmed the pathogenicity of the identified variant, we utilized in-silico tools and cross-referenced various databases to confirm its novelty. Additionally, we conducted a co-segregation study and verified the presence of the variant in the parents of the affected patients through Sanger sequencing.

**Results:**

In our study, we identified a novel start-loss mutation (c.2T > A, p.Met1Lys) in the SLC29A3 gene, which was found in both of two patients. Co-segregation analysis using Sanger sequencing confirmed that this variant was inherited from the parents. To evaluate the potential pathogenicity and novelty of this mutation, we consulted various databases. Additionally, we employed bioinformatics tools to predict the three-dimensional structure of the mutant SLC29A3 protein. These analyses were conducted with the aim of providing valuable insights into the functional implications of the identified mutation on the structure and function of the SLC29A3 protein.

**Conclusion:**

Our study contributes to the expanding body of evidence supporting the association between mutations in the *SLC29A3* gene and H syndrome. The molecular analysis of diseases related to *SLC29A3* is crucial in understanding the range of variability and raising awareness of H syndrome, with the ultimate goal of facilitating early diagnosis and appropriate treatment. The discovery of this novel biallelic variant in the probands further underscores the significance of utilizing genetic testing approaches, such as WES, as dependable diagnostic tools for individuals with this particular condition.

## Introduction

H syndrome, also referred to as histiocytosis-lymphadenopathy plus syndrome or PHID, is an autosomal recessive disorder characterized by abnormal proliferation of histiocytes. This condition is caused by mutations in the *SLC29A3* gene located on chromosome 10q22.1. The *SLC29A3* gene encodes the human equilibrative nucleoside transporter (hENT3) protein [1]. ENT3 is a member of the SLC29-equilibrating nucleoside transporter family, a group of proteins involved in transporting nucleosides across cellular membranes. ENT3 is widely expressed in various tissues throughout the body. Its cellular expression is particularly significant in the membranes of lysosomes and mitochondria. These organelles play crucial roles in cellular metabolism and homeostasis, and the presence of ENT3 in their membranes suggests its involvement in nucleoside transport within these compartments [2].

ENT3 facilitates the passive, sodium-independent transport of nucleobases, nucleotides, and nucleotide analogs across the lysosomal membrane, allowing their movement from the lysosome to the cytoplasm. Similarly, it aids in the transport of these molecules across the inner mitochondrial membranes. These processes are essential for maintaining an adequate cytoplasmic pool of nucleosides necessary for various cellular pathways and functions [3, 4]. Mutations in SLC29A3 can lead to impaired phagocytosis, which in turn causes an excessive inflammatory response and abnormal proliferation of histiocytes, contributing to the clinical features observed in H syndrome [1, 5]. Furthermore, these mutations can hinder the transportation of nucleosides, resulting in the accumulation of nucleosides within the cells. This disruption in nucleoside transport can have significant impacts on cellular processes and contribute to the pathogenesis of H syndrome.

H syndrome can arise from various types of mutations in the *SLC29A3* gene, including nonsense, missense, compound (involving multiple genetic alterations), or deletion mutations. The presence of different mutation types in *SLC29A3* may partially explain the extensive variability observed between different families affected by H syndrome. The diverse array of genetic alterations in *SLC29A3* can contribute to the wide range of clinical presentations and manifestations observed in individuals with H syndrome [6, 7].

H syndrome was initially described by Molho-Pessach et al. in 2008, and it was named as such because many of its clinical features start with the letter “H” [8]. The syndrome is characterized by several hallmark manifestations, including symmetrical hyperpigmentation of the skin accompanied by hypertrichosis and sclerodermatous induration, hyperglycemia or insulin-dependent diabetes mellitus, hearing loss, hypergonadotropic hypogonadism with scrotal masses, short stature, gynecomastia, hepatosplenomegaly, hallux valgus, and heart anomalies. It is currently recognized as a type of histiocytosis that affects multiple organs [9].

This study aims to elucidate the molecular basis of H syndrome in two affected individuals from a single Iranian family by employing WES methodology. WES specifically targets the sequencing of exons, which represent the crucial protein-coding regions of the genome. By examining the entire exome, the study aimed to identify any genetic variations or mutations that could be responsible for the development of H syndrome in the two patients.

## Materials and methods

### Patients

In this study, we conducted an examination of 2 patients, a 16-year-old girl and her 8-year-old brother, who was born to consanguineous parents. The both patients presented with fever, skin hyperpigmentation, hearing loss, organ enlargement, and diabetes. The documentation of this study conforms to the Declaration of Helsinki protocols, was approved by the Ethical Research Committee of the Golestan University of Medical Sciences (No. IR.GOUMS.REC.1401.227), and informed consent was given by the parents of the children for publication of photographs and a description of the children’s presentation.

### DNA extraction

For genetic testing, whole blood samples were collected from patients, their normal sister and parents. Genomic DNA was extracted from blood samples by Yekta Tajhiz DNA extraction kit (Cat. No., FABGK001) according to the manufacturer’s instructions. DNA concentrations were calculated using a NanoDrop spectrophotometer.

### Whole-exome sequencing

The DNA extracted from the sample was fragmented and tagged with barcodes, followed by a solution-based hybridization step using the Agilent SureSelect Human All Exon V7 Plus probe set. Subsequently, the exome sequence was obtained using next-generation sequencing on the Illumina NovaSeq 6000 platform. The sequencing process achieved an average target coverage of 100X, ensuring that 90–95% of bases had a minimum coverage of 20X.

Analysing WES data involved four steps: [1] The Burrows-Wheeler alignment software (version 0.7.5a) was employed to align cleaned reads to the human reference genome (hg19). [2] Duplicate reads were identified using Picard (version 2.25). [3] Realignment of insertions and deletions was conducted with the Genome Analysis Toolkit (GATK, version 2.4–9). [4] Variants were identified using both GATK and Samtools programs. The results were combined and annotated with various databases including dbSNP, the 1000 Genomes Project, and ClinVar. Annotation analysis of the detected variants was carried out using ANNOVAR software.

### In-Silico analysis identified variant

To assess the potential disease-causing nature of the identified variant, we conducted an in-silico analysis. This analysis involved the utilization of computational tools, including ACMG [10], CADD [11], Mutation Taster [12], Polyphen-2 [13], SIFT [14], Panther [15], Fathmm [16], MUpro [17], and I-Mutant [18]. Additionally, we manually evaluated the most significant variations to confirm their correlation with actual clinical results.

### Sequencing and Co-segregation study

The co-segregation analysis aimed to investigate the transmission of the identified genetic variation across generations. By evaluating the co-segregation of variants with the disease phenotype, we can determine their potential role in disease onset. During the initial stage, polymerase chain reaction (PCR) amplification was carried out by utilizing specialized primers designed to target the specific site of the identified variation within the *SLC29A3* gene. Sanger sequencing was then conducted to validate and confirm the presence of the variation in the probands and their parents. Finally, the obtained sequence chromatograms were assessed using the Codon Code Aligner software.

### Prediction of protein three-Dimensional structure

In our study, we employed the I-TASSER server (https://zhanglab.ccmb.med.umich.edu/I-TASSER/) to generate three-dimensional (3D) models of both the mutant and wild-type forms of SLC29A3. This server utilizes the Protein Data Bank (PDB) to identify similar sequences that correspond to different regions of the input sequence. These sequences are then combined to construct a comprehensive 3D structure, which is further refined using molecular simulations and modeling techniques to improve stability and optimize energy. To visualize the resulting model, we utilized the UCSF Chimera software (https://www.cgl.ucsf.edu/chimera/).

### Study of protein-protein interactions

To explore protein-protein interactions associated with SLC29A3, we employed the online tool STRING (https://string-db.org/). This resource incorporates information from various datasets, encompassing gene fusion, co-expression patterns, functional annotations, and experimental findings. It enables the prediction of potential protein partners that may interact with SLC29A3. Each identified interacting protein receives a comprehensive score between 0 and 1, reflecting the strength of the interaction. A score of 0 indicates limited interaction, whereas a score of 1 indicates substantial interaction.

### Conservation analysis

To explore the evolutionary conservation of the amino acid sequence in the SLC29A3 protein, we employed the Clustal Omega web server. [19]. Our objective was to assess the protein sequence and determine its conservation across different species. We obtained SLC29A3 protein sequences from various species, such as the tropical clawed frog (Xenopus tropicalis), red junglefowl (Gallus gallus), house mouse (Mus musculus), brown rat (Rattus norvegicus), chimpanzee (Pan troglodytes), rhesus macaque (Macaca mulatta), humans (Homo sapiens), domestic cattle (Bos taurus), and dog (Canis lupus familiaris), from NCBI. These sequences were appropriately formatted for alignment using Clustal Omega.

## Results

### Clinical finding

The studied families are of Iranian origin located in Golestan province (Northeast Iran). They were the offspring of healthy consanguineous parents. The pregnancy and delivery of these patients were uncomplicated and did not present any noteworthy issues. Importantly, there was no reported history of birth defects or spontaneous abortions within the family.

**Fig. 1:**
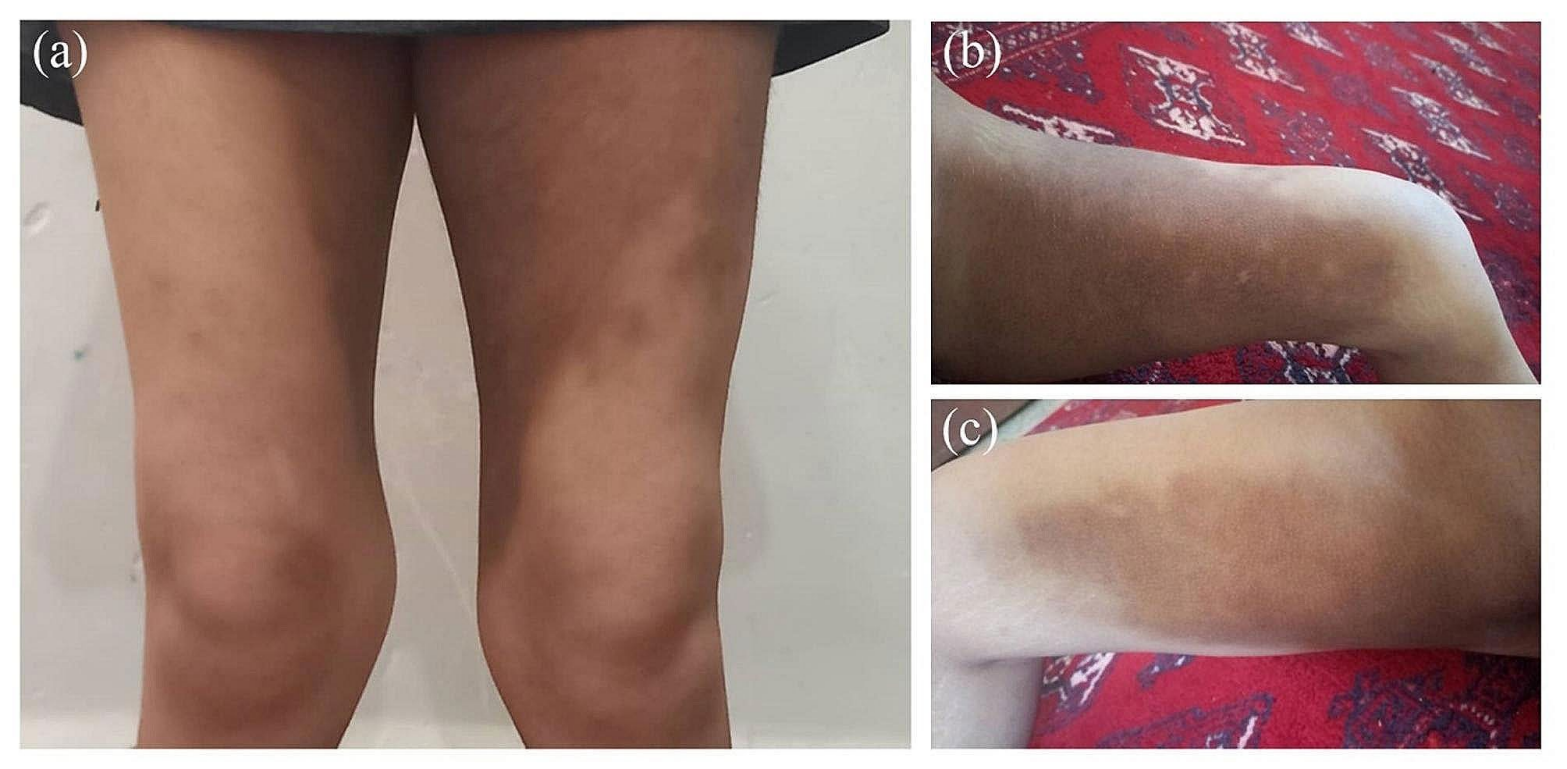
The clinical findings of two patients with H syndrome. (**a** and **b**) Proband 1, bilateral dark hyperpigmented with hypertrichosis on the front side of thighs and groin. (**c**) Proband 2, hyperpigmented, hypertrichotic skin lesions on the groin. It is crucial to note that H syndrome patients may present with a variety of symptoms and manifestations beyond those depicted in this figure

The patient 1 involves a 16-year-old girl. She exhibited symptoms of H syndrome from 9 year of age, including fever, widespread organ involvement, and additional manifestations affecting the skin, joints, hearing, and the endocrine system. Notably, she experienced bilateral hearing loss and exhibited cardiac, kidney dysfunction, and diabetes. Patient 2 was the younger brother (8-year-old) of patient 1. He exhibited symptoms of H syndrome at 6 year of age, including fever, lymphadenopathy, and organ enlargement, and skin hyperpigmentation, unilateral hearing loss, indicative of an auto inflammatory, endocrine, and cutaneous disorder, and diabetes. Figure 1 shows skin hyperpigmentation and hypertrichosis in both patients, which is more severe in patient 1. The clinical and demographic data are summarized in Table 1.

**Table 1:** Summary of clinical and demographic characteristics of children with H syndrome

Characteristic	Patient 1	Patient 2
Age (years)	15	8
Gender	Female	Male
Height (cm)	146	127
Weight (kg)	43	24
Age of onset (years)	1	6
Hearing loss	Severe (left), profound (right)	Mild (left)
ESR (mm/h)	103	47
Cutaneous hyperpigmentation	+	+
Hypertrichosis	+	+
Heart anomalies	Cardiac mass	-
Renal impairment	Decreased radiotracer uptake moderate hydronephrosis (left)	-
IDDM	+	+
CRP (mg/dL)	44	31
Thyroid nodule	Thyroid colloid cyst	-

ESR: erythrocyte sedimentation rate. IDDM: insulin-dependent diabetes mellitus. CRP: C-reactive protein

### Clinical genetic testing

**Fig. 2:**
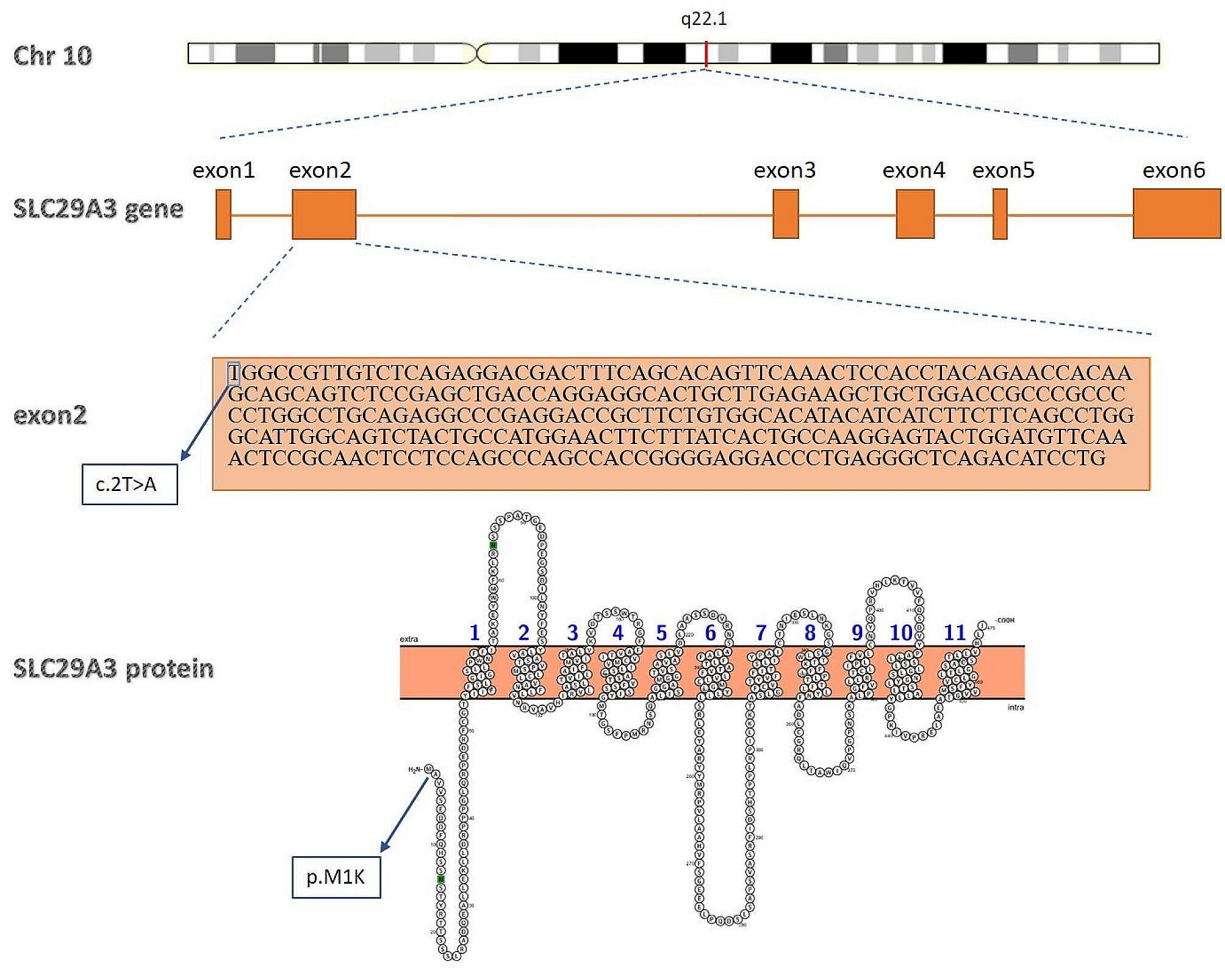
A graphical representation of the *SLC29A3* gene structure and mutation position

WES was performed on both patients, considering their phenotypes and initial diagnosis. The genetic analysis led to the identification of a novel single nucleotide start loss variant in the *SLC29A3* gene, specifically the c.2T > A (p.M1K) NM_018344.6 variant, which was found in both patients. This particular variant has been associated with the manifestation of H syndrome. Figure 2 illustrates the genomic position of the c.2T > A mutation within the *SLC29A3* gene, providing details at both the chromosomal and nucleotide levels.

### Co-segregation study

**Fig. 3:**
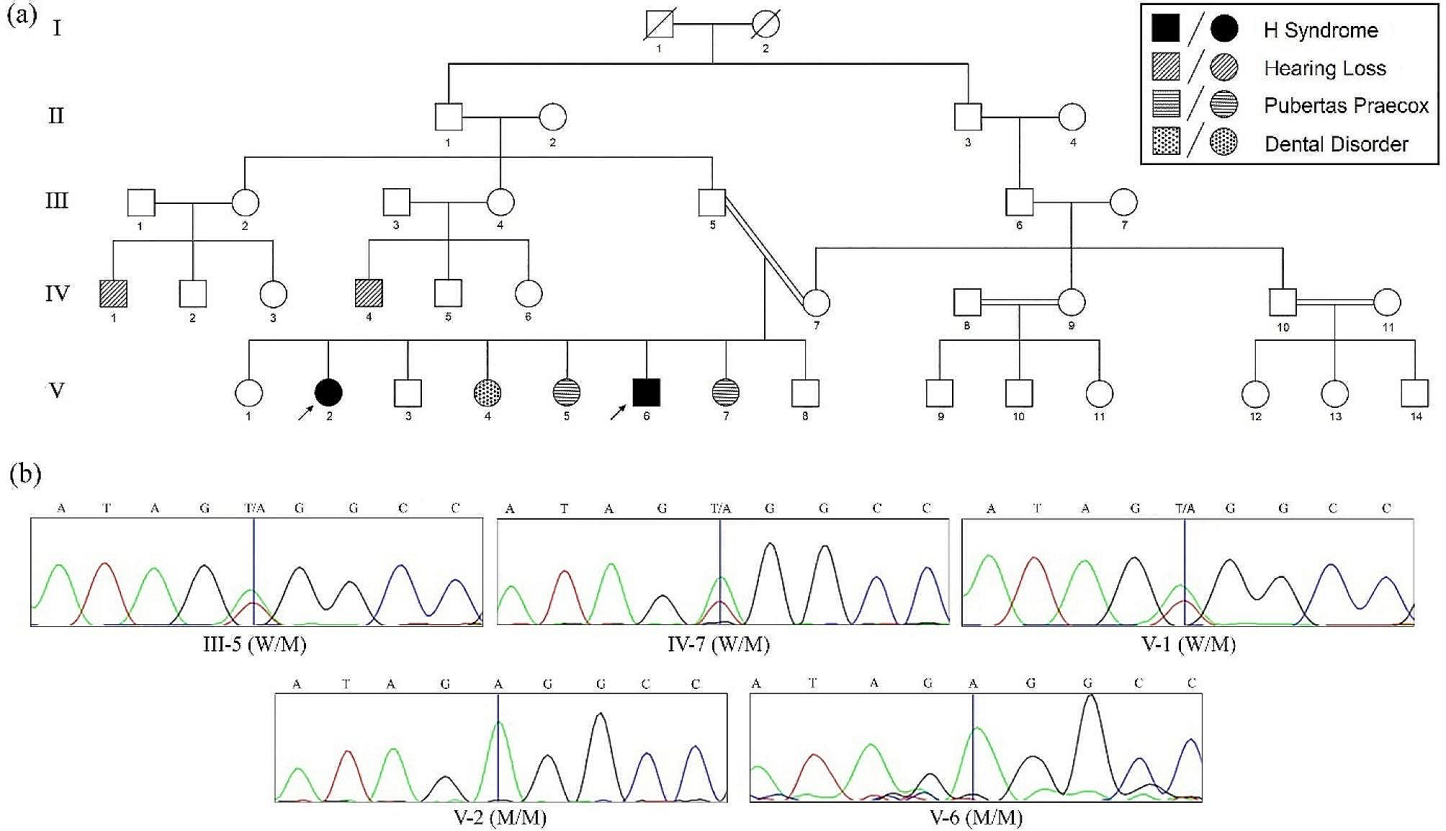
(**a**) Pedigree of family with H syndrome. Two children with H syndrome. The affected siblings are indicated with filled symbols. (**b**) Sanger sequencing chromatogram confirmation of WES showing a homozygous start loss Varian c.2T > A in the 16-yr old proband (marked with an arrow) and her 8-yr old brother. The mother, father and sister, who are healthy, are heterozygous for the mutation. (W/M: wild/mutant. M/M: mutant/mutant)

To confirm the presence and inheritance pattern of the mutation, Sanger sequencing was conducted on the probands, their unaffected sibling, and their parents. The analysis revealed that the unaffected sibling and parents were carriers of the variant in a heterozygous state, while both patients were homozygous for the identified variant. Figure 3 displays the pedigree of the family affected by H syndrome, along with the sequencing chromatogram obtained from the WES data.

### Pathogenicity and protein stability analysis

The identified variant, c.2T > A, is novel and has not been previously reported. In order to evaluate its potential pathogenicity and its effect on protein stability, we utilized multiple prediction tools. The results of this in silico analysis, conducted using various computational predictors including ACMG, Mutation Taster, CADD, Polyphen-2, SIFT, Panther, Fathmm, MUpro, and I-Mutant, are summarized in Table 2.

**Table 2:** Results of in silico analysis using computational predictors for the c.2T > A mutation in *SLC29A3*

Gene	Mutation	Protein			ACMG	CADD	Mutation Taster	Polyphon-2	panther	MUpro	I-Mutant 2	fathmm
SLC29A3	c.2T > A		p.M1K	Likely Pathogenic		23.2	Disease causing	BENIGN (score:0.261)	probably damaging (Pdel: 0.78)	Decrease (DDG: 2.23)	Decrease (-1.22)	Tolerated

### Tertiary structure, and protein–protein Interaction

**Fig. 4:**
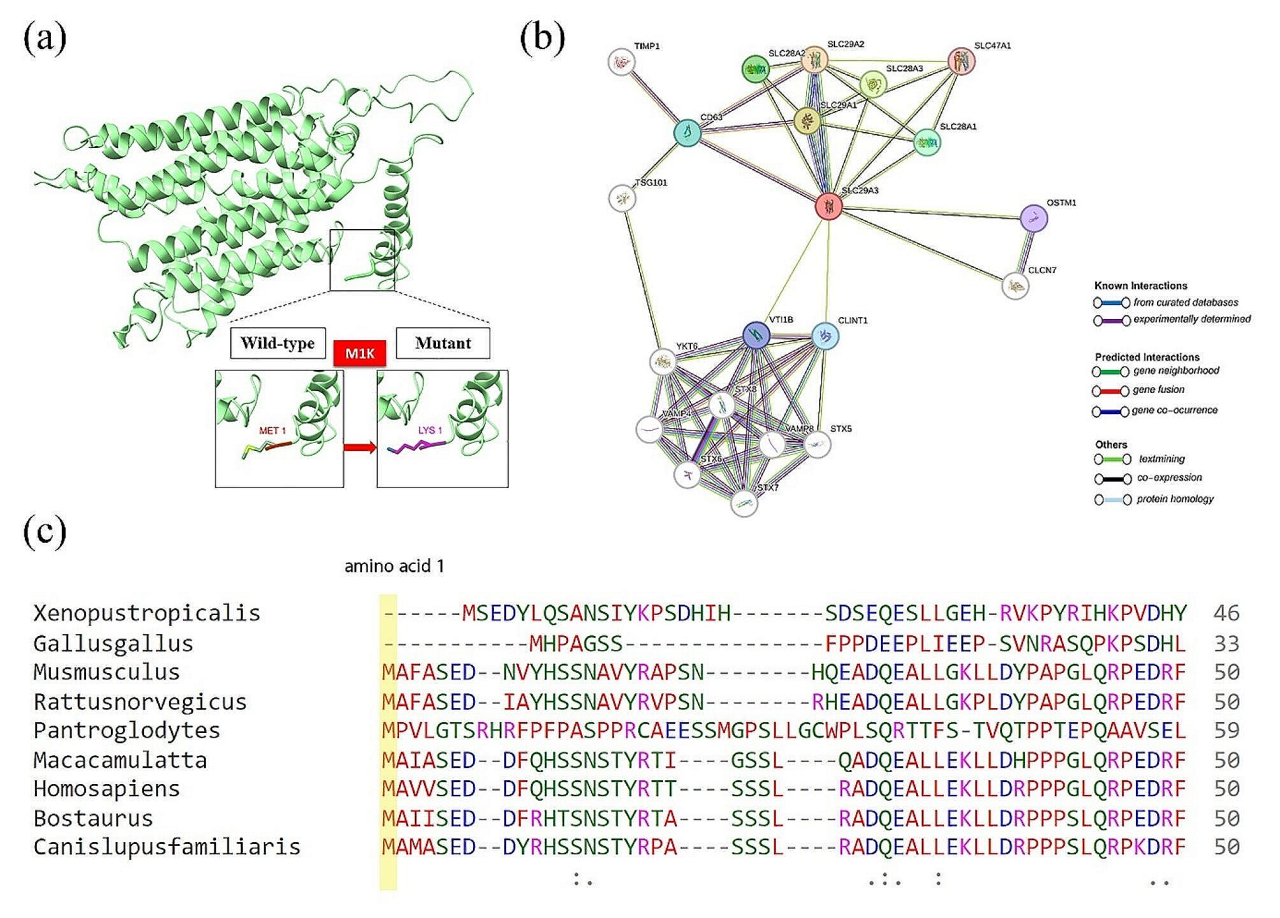
(**a**) 3D structure of the SLC29A3 protein. (**b**) Prediction of protein-protein interaction based on STRING data, visualized by NetworkX. (**c**) Evolutionary conservation analysis of p.Met mutation on SLC29A3 using Clustal Omega tool

Figure 4a shows the 3D structure of the SLC29A3 protein, including both the wild-type form and the mutant form with the p.M1K variation. We used the String tool to find the interacting proteins of SLC29A3 that were closest in proximity. The first shell interactors of SLC29A3 are shown in Fig. 4b and include the following: SLC29A2, SLC29A1, SLC28A3, SLC28A2, SLC28A1, CD63, CLINT1, VTI1B, OSTM1, and SLC47A1. Among these proteins, it is predicted that SLC29A2, with a score of 0.925, has the highest level of interaction with SLC29A3. Second shell interactors are also present, including YKT6, VAMP4, VAMP8, STX5, STX6, STX7, and STX8.

### Amino acids conservation

The amino acid 1 (Methionine), which is the point of variation, was shown to be substantially conserved in all the investigated species when the SLC29A3 protein sequence was aligned across several species using the Clustal Omega tool. According to this finding, the residue is critical to the protein’s evolutionary and functional history. In Fig. 4c the outcomes are highlighted.

## Discussion

Molho-Pessach et al. first characterized H syndrome in a study of 10 patients from 6 consanguineous Arab families [20], who had hyperpigmentation, hypertrichosis, and indurated cutaneous patches in the middle and lower parts of their bodies. In addition, these patients experienced hepatosplenomegaly and sensorineural hearing loss [21]. The clinical observations of our patients were in agreement with the previously reported cases. Bolze et al. also observed that sensorineural hearing loss and hepatosplenomegaly are present in approximately half of the individuals diagnosed with H syndrome [22]. Numerous studies have consistently reported that hyperpigmentation and hypertrichosis are the most common clinical features observed in individuals with H syndrome, and they are often regarded as pathognomonic signs of the condition [7, 21]. However, Bloom et al. faced challenges in diagnosing their patients with H syndrome and were only able to achieve a diagnosis through WES. They attributed the diagnostic difficulties to the relatively recent description of the syndrome and the limited number of cases previously published [23].

Similarly, in Iran, there have been only a few reported cases of H syndrome, which can be attributed to the rarity of the syndrome and the overlapping clinical manifestations it shares with other conditions. As a result, diagnosing H syndrome in Iran and other regions with limited reported cases may be more challenging due to the lack of familiarity and awareness of the syndrome among healthcare professionals.

To date, more than 50 pathogenic or likely pathogenic variants with H syndrome have been reported (Table 3), predominantly from the Arab populations of Egypt, Iran, Tunisia, Israel, Turkey, Syria, and Morocco [24]. The majority of these patients are diagnosed in early life, typically due to the presence of either skin-related changes or diabetes mellitus. The average age at diagnosis is around 17 years [25]. According to the study conducted by El-Darouti et al., in a patient with a combination of hepatosplenomegaly, hearing loss, heart anomalies, hypertrichosis, hypogonadism, and short stature, H syndrome should be considered as a potential differential diagnosis [26].

**Table 3:** A summary of the mutations reported in SLC29A3 gene

Nucleotide change	Protein change	Exon/Intron	ACMG classification	Condition	(Reference/ClinVar ID)
c.243del	p.Lys81fs	Exon 2	Pathogenic	H syndrome	[22]
c.308_309del	p.Tyr102_Phe103insTer	Exon 3	Pathogenic	H syndrome	[27]
c.300 + 1G > A	NA	Exon 2	Pathogenic	H syndrome	[28]
c.73 C > T	p.Arg25Ter	Exon 2	Pathogenic	Histiocytosis-lymphadenopathy plus syndrome	(ID: 212,200)
c.300 + 1G > C	NA	Exon 2	Pathogenic	not provided	(ID: 427,021)
c.347 T > G	p.Met116Arg	Exon 3	Likely Pathogenic	H syndrome	[28]
c.940del	p.Tyr314ThrfsTer91	Exon 6	Likely Pathogenic	H syndrome	[28]
c.1309G > A	p.Gly437Arg	Exon 6	Pathogenic	H syndrome	[28]
c.1330G > T	p.Glu444Ter	Exon 6	Pathogenic	H syndrome	[28]
c.1346 C > G	p.Thr449Arg	Exon 6	Likely Pathogenic	SLC29A3-related condition	[28]
c.479G > A	p.Trp160Ter	Exon 4	Pathogenic	H syndrome	(ID: 573,984)
c.607 T > C	p.Ser203Pro	Exon 4	Uncertain Significance	Dysosteosclerosis	[29]
c.1346 C > G	p.Thr449Arg	Exon 6	Likely Pathogenic	SLC29A3-related condition	[29]
c.714_715invTG	p.Val239Ile	Exon 6	Likely Pathogenic	Histiocytosis-lymphadenopathy plus syndrome	(ID: 300,363)
c.1001 A > G	p.Asn334Ser	Exon 6	Benign	Histiocytosis-lymphadenopathy plus syndrome	(ID: 300,368)
c.1045delC	p.Leu349Serfs	Exon 6	Pathogenic	H syndrome	[21]
c.1087 C > T	p.Arg363Trp	Exon 6	Pathogenic	H syndrome	[30]
c.1088G > A	p.Arg363Gln	Exon 6	Likely Pathogenic	H syndrome, Faisalabad histiocytosis	[30]
c.1228 C > T	p.Gln410Ter	Exon 6	Pathogenic	H syndrome	(ID: 130,338)
c.1279G > A	p.Gly427Ser	Exon 6	Pathogenic	H syndrome	[31]
c.307-308delTT	p.Phe103Terfs	Exon 3	Pathogenic	H syndrome	[32]
c.101_104dup	p.Leu36fs	Exon 2	Pathogenic	H syndrome	(ID:1,366,169)
c.1045del	p.Leu349fs	Exon 6	Pathogenic	H syndrome	(ID:566)
c.1077_1084del	p.Asp359fs	Exon 6	Likely Pathogenic	H syndrome	(ID:2,721,497)
c.122del	p.Pro41fs	Exon 2	Pathogenic	H syndrome	(ID:915,355)
c.1294del	p.Leu432fs	Exon 6	Likely Pathogenic	H syndrome	(ID:1,993,275)
c.1295del	p.Leu432fs	Exon 6	Likely Pathogenic	H syndrome	(ID:1,993,276)
c.139G > T	p.Glu47Ter	Exon 2	Pathogenic	H syndrome	(ID:862,781)
c.243del	p.Lys81fs	Exon 2	Pathogenic	not provided	(ID:636,986)
c.269_275del	p.Thr90fs	Exon 2	Pathogenic	H syndrome	(ID:1,069,036)
c.300 + 2T > C	NA	Exon 2	Pathogenic	H syndrome	(ID:1,451,137)
c.382_383del	p.Arg128fs	Exon 3	Pathogenic	H syndrome	(ID:2,086,492)
c.443del	p.Val148fs	Exon 4	Pathogenic	H syndrome	(ID:1,390,366)
c.479G > A	p.Trp160Ter	Exon 4	Pathogenic	H syndrome	(ID:573,984)
c.59_60dup	p.Ser21fs	Exon 2	Likely Pathogenic	H syndrome	(ID:2,783,465)
c.67_70del	p.Leu24fs	Exon 2	Likely Pathogenic	H syndrome	(ID:2,760,413)
c.777 C > A	p.Tyr259Ter	Exon 6	Likely Pathogenic	H syndrome	(ID:2,788,367)
c.919dup	p.Ser307fs	Exon 6	Likely Pathogenic	H syndrome	(ID:2,750,513)
c.940del	p.Tyr314fs	Exon 6	Likely Pathogenic	H syndrome	(ID:567)
c.963del	p.Ile322fs	Exon 6	Likely Pathogenic	H syndrome	(ID:1,354,691)
c.984del	p.Asn329fs	Exon 6	Pathogenic	H syndrome	(ID:664,261)
c.1 + 2T > G	NA	intron 1	Likely Pathogenic	H syndrome	(ID:2,136,876)
c.1077_1084del	p.Asp359fs	Exon 6	Likely Pathogenic	H syndrome	(ID:2,721,497)
c.2–4 A > G	NA	Exon 2	Likely Pathogenic	H syndrome	(ID:2,441,776)
c.400 C > T	p.Arg134Cys	Exon 4	Likely Pathogenic	H syndrome	(ID:1,693,224)
c.610 + 1G > A	NA	Exon 4	Pathogenic	H syndrome	(ID:1,484,112)
c.971 C > T	p.Pro324Leu	Exon 6	Likely Pathogenic	H syndrome	(ID:963,811)
c.611-1G > T	NA	Exon 5	Pathogenic	H syndrome	(ID:659,679)
c.303_320dupCTACTTTGAGAGCTACCT	p.Tyr102_Leu107dup	Exon 3	Likely Pathogenic	dysosteosclerosis	[33]
c.1157G > A	p. Arg386Gln	Exon 6	Uncertain Significance	SLC29A3-related condition	[34]
p.Leu298del	p. Leu298fs	Exon 6	Uncertain Significance	SLC29A3-related condition	[34]
c.416T > C	p.Leu139Pro	Exon 4	Uncertain Significance	H syndrome	[35]
c.607 T > C	p.Ser203Pro	Exon 4	Uncertain Significance	Osteopetrosis, intermediate and platyspondyly	[36]
c.1157G > A	p.Arg386Gln	Exon 6	Uncertain Significance	Osteopetrosis, intermediate and platyspondyly	[36]
c.2T > A	p.Met1Lys	Exon 2	Likely Pathogenic	H syndrome	This study

The H syndrome is caused by the presence of mutations in both alleles of the SLC29A3 gene, which is located on chromosome 10q22 and encodes the hENT3 protein. This protein plays a vital role in transporting nucleosides across cellular membranes, thereby contributing to the regulation of the cytoplasmic pool of nucleosides necessary for nucleotide synthesis, including salvage pathways [4]. The histiocytes and macrophages rely on the salvage pathways for nucleotide synthesis since they have a deficiency in endogenous nucleotide production. The disruption of nucleoside reserves is believed to affect the intracellular synthesis rate of extracellular matrix proteins produced by histiocytes. This alteration ultimately leads to significant subcutaneous and dermal fibrosis, which is considered the primary pathological process in H syndrome [37].

As mentioned earlier, mutations in the *SLC29A3* gene, including missense, nonsense, and deletion mutations, lead to various medical manifestations ranging from mild to severe, which are known as the same entity termed H syndrome. Currently, over 50 pathogenic or likely pathogenic mutations have been documented in the *SLC29A3* gene [23, 32], but so far, no clear correlation between specific genotypes and phenotypes has been recognized [38]. Various allelic disorders have been identified, including pigmented hypertrichosis with insulin-dependent diabetes mellitus (PHID) syndrome, familial histiocytosis syndrome (FHS), Faisalabad histiocytosis (FHC), familial sinus histiocytosis with massive lymphadenopathy (FSHML; also named familial Rosai-Dorfman disease), and some types of dysosteosclerosis. However, due to the fact that H syndrome encompasses the majority of these clinical features and all of these disorders share identical mutations in the *SLC29A3* gene, they are now considered to be the same entity [4, 21, 32, 39].

In this study, we identified a novel homozygous missense mutation in exon 2, c.2T > A p.(Met1Lys), on *SLC29A3* (NM_018344.6) that results in H syndrome. To validate our findings, we performed Sanger sequencing, which not only confirmed the presence of the identified mutation but also demonstrated its co-segregation with the mutation in the parents. As the identified mutation in this study was a novel one, its potential pathogenicity was assessed using bioinformatics tools. These computational tools analyse various aspects of the mutation, such as its location within the gene, the predicted impact on protein structure and function, and its conservation across different species. Based on the results from these bioinformatics analyses, the pathogenicity of the mutation was evaluated. It is worth noting that variants at translational starts sites are disruptive as they prevent protein expression. These variants are often classified as pathogenic, unless an alternative translational start is shown to produce a functional isoform to rescue protein expression. the c.2T > A start codon mutation is expected to result in a complete loss of function of the SLC29A3-encoded ENT3 protein. This would likely have significant cellular and physiological consequences due to the important role of ENT3 in nucleoside transport. Further experimental validation would be needed to fully characterize the impact of this mutation. Ultimately, the findings from the bioinformatics assessment were confirmed, establishing the mutation as pathogenic and contributing to the development of the observed H syndrome. The defining characteristic of the syndrome is the presence of cutaneous hyperpigmentation and hypertrichosis, primarily observed on the lower limbs, typically manifesting in the first or second decade of life [35].

Patient 1 and Patient 2 both exhibited characteristic symptoms of the H syndrome, including hyperpigmented indurated plaques with hypertrichosis on their lower limbs. Additionally, both patients presented with short stature, insulin-dependent diabetes mellitus (IDDM), and elevated inflammatory markers. Patient 1 also experienced bilateral hearing loss, cardiac and kidney dysfunction, as well as diabetes, while Patient 2 had unilateral hearing loss, short stature, and diabetes. The clinical features of the older sister (Patient 1) were more pronounced and well-defined. The presented pedigree highlights the presence of phenotypic variability within affected individuals, suggesting that clinical features may continue to evolve over time. It is worth noting that the observed variation could be influenced by the age difference between the sister and her brother. It is important to note that our study had certain limitations. First, we did not conduct functional studies to evaluate the clinical features of mutant animal models with c.2T > A in SLC29A3 gene, which could provide more robust evidence for the pathogenicity of the identified mutation. Second, we did not evaluate the level of SLC29A3 protein expression using techniques such as western blot analysis, which could have been helpful in determining whether the mutation resulted in the production of truncated protein or complete loss of function. Furthermore, WES has limitations, as it cannot detect certain types of genetic variations, such as chromosomal structural rearrangements, copy number variations (CNVs), mosaicism, changes in repetitive DNA sequences, epigenetic modifications, alterations in mitochondrial genes, or variations in non-coding regions. To enhance our understanding of the precise role of the *SLC29A3* gene in H syndrome, additional research and genetic investigations are crucial. These endeavors will contribute to a more comprehensive comprehension of the condition.

## Conclusion

This research demonstrates that a specific genetic mutation, c.2T > A, which causes the loss of a start codon in a homozygous state, leads to a range of clinical symptoms associated with H syndrome. Alongside the presence of different variations of the gene, it appears that the diverse clinical manifestations strongly support the idea of the *SLC29A3* gene having multiple effects and variable expression, possibly indicating the participation of other genes. In essence, these findings highlight the effectiveness of whole-exome sequencing as a valuable approach for identifying genes associated with genetic disorders in humans.

## Data Availability

The datasets generated and/or analyzed during the current study are available in the ClinVar repository, ClinVar accession number: SCV004697500.
